# *catena*-Poly[[diaqua­manganese(II)]-di-μ-pyridine-3-sulfonato-κ^2^ *N*:*O*;κ^2^ *O*:*N*]

**DOI:** 10.1107/S1600536808012282

**Published:** 2008-05-03

**Authors:** Zhi-Hui Qiu, Fu-Pei Liang, Qing-Feng Ruan, Shan-Rong Zhao

**Affiliations:** a College of Chemistry and Chemical Engineering, Guangxi Normal University, Guilin 541004, People’s Republic of China; b Faculty of Earth Sciences, China University of Geosciences, Wuhan 430074, People’s Republic of China; c Department of Resources and Environmental Engineering, Guilin University of Technology, Guilin 541004, People’s Republic of China

## Abstract

In the title polymeric complex, [Mn(C_5_H_4_NO_3_S)_2_(H_2_O)_2_]_*n*_, the Mn atom is located on a centre of inversion and is coordinated by two O atoms and two N atoms derived from four different pyridine-3-sulfonate (pySO_3_) ligands, and two O atoms derived from two water mol­ecules in a distorted *trans*-N_2_O_4_ octa­hedral geometry. The metal atoms are bridged by the pySO_3_ ligands to form a one-dimensional chain. The chains are further connected into a three-dimensional architecture *via* hydrogen bonds.

## Related literature

For related structures, see: Brodersen *et al.* (1980 ▶); Chandrasekhar (1977 ▶); Cotton *et al.* (1992*a* ▶,*b* ▶); Mäkinen *et al.*(2001 ▶); Van der Lee & Barboiu (2004 ▶); Walsh & Hathaway (1980 ▶). For a description of the Cambridge Structural Database, see: Allen (2002 ▶).
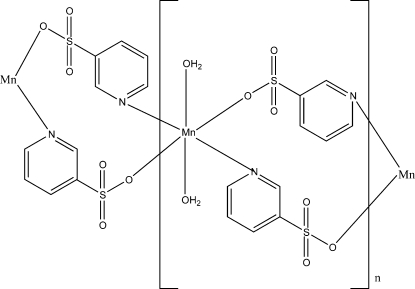


## Experimental

### 

#### Crystal data

[Mn(C_5_H_4_NO_3_S)_2_(H_2_O)_2_]
*M* *_r_* = 407.28
Monoclinic, 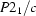

*a* = 7.6299 (13) Å
*b* = 13.201 (2) Å
*c* = 7.2714 (12) Å
β = 96.516 (3)°
*V* = 727.7 (2) Å^3^
*Z* = 2
Mo *K*α radiation
μ = 1.24 mm^−1^
*T* = 294 (2) K
0.24 × 0.22 × 0.18 mm

#### Data collection

Bruker SMART CCD area-detector diffractometer
Absorption correction: multi-scan (*SADABS*; Bruker, 1998 ▶) *T*_min_ = 0.755, *T*_max_ = 0.808
4034 measured reflections
1485 independent reflections
1301 reflections with *I* > 2σ(*I*)
*R*_int_ = 0.022

#### Refinement

*R*[*F* ^2^ > 2σ(*F* ^2^)] = 0.026
*wR*(*F* ^2^) = 0.069
*S* = 1.06
1485 reflections
106 parameters
H-atom parameters constrained
Δρ_max_ = 0.24 e Å^−3^
Δρ_min_ = −0.37 e Å^−3^

### 

Data collection: *SMART* (Bruker, 1998 ▶); cell refinement: *SAINT* (Bruker, 1998 ▶); data reduction: *SAINT*; program(s) used to solve structure: *SHELXS97* (Sheldrick, 2008 ▶); program(s) used to refine structure: *SHELXL97* (Sheldrick, 2008 ▶); molecular graphics: *SHELXTL* (Sheldrick, 2008 ▶); software used to prepare material for publication: *SHELXTL*.

## Supplementary Material

Crystal structure: contains datablocks I, global. DOI: 10.1107/S1600536808012282/gk2137sup1.cif

Structure factors: contains datablocks I. DOI: 10.1107/S1600536808012282/gk2137Isup2.hkl

Additional supplementary materials:  crystallographic information; 3D view; checkCIF report

## Figures and Tables

**Table 1:** Hydrogen-bond geometry (Å, °)

*D*—H⋯*A*	*D*—H	H⋯*A*	*D*⋯*A*	*D*—H⋯*A*
O4—H4*B*⋯O2^i^	0.89	1.91	2.786 (2)	168
O4—H4*A*⋯O1^ii^	0.89	1.90	2.778 (2)	169

Symmetry codes: (i) 
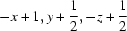
; (ii) 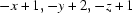.

**Fig. 1.**
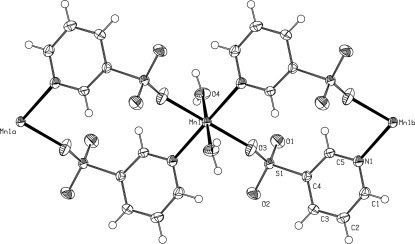
The atom-numbering scheme and 50% probability displacement ellipsoids for the title compound. The Mn atom is located at a center of inversion. H atoms are shown as small spheres of arbitrary radii [symmetry code: (a) -1 + x,y,z].

**Fig. 2.**
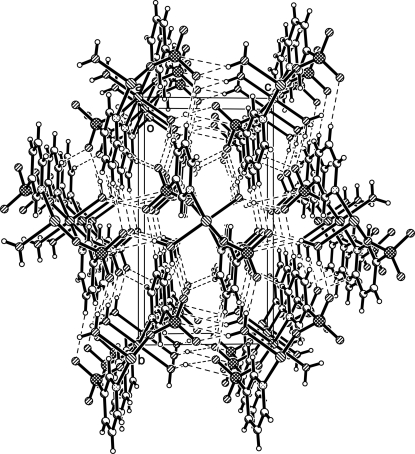
Crystal packing of the title compound viewed approximately down the a-direction showing the hydrogen bonding interactions as dashed lines.

## supplementary crystallographic information

### Comment

Structures of complexes or salts based on pyridinium-3-sulfonate are not numerous in the Cambridge Structural Database (CSD; Version 5.25; Allen, 2002). Some six-coordinate metal complexes with pyridine-3-sulfonate (pySO_3_) ligands that are closely related to the title complex have been reported (Walsh & Hathaway, 1980; Cotton *et al.*, 1992*a*). Other pySO_3_ complexes are also available (Brodersen *et al.*, 1980; Cotton *et al.*, 1992*b*; Mäkinen *et al.*, 2001; van der Lee & Barboiu, 2004), as well as that of the pySO_3_H acid (Chandrasekhar, 1977). There are two structures of the [*M*(pySO_3_)_2_(H_2_O)_2_] type in the CSD. One of them is isostructural with the title compound (Walsh & Hathaway, 1980; Cotton *et al.*, 1992*a*) and the other structure is a two-dimensional coordination polymer (Brodersen *et al.*,1980).

The Mn atom is located on a centre of inversion and is six-coordinated by two N atoms and two O atoms derived from four different pySO_3_, and two O atoms derived from two water molecules (Fig. 1). The resulting *trans*-N_2_O_4_ donor set defines a distorted octahedral environment for Mn. The bond angles deviate considerably from 90°; those derived from the bulkier groups deviate by nearly 6°. The Mn—O(water) distance of 2.1681 (15) Å and Mn—O(pySO_3_) distance of 2.1772 (15) Å are in the usual range.The Mn—N distance is also in the usual range for pyridine-like ligands.

The metal ions are bridgeding pySO_3_ anions to form a chain. In the crystal structure, chains are linked into a 3-D architecture *via* hydrogen bonding interactions (Table 1 & Fig. 2).

### Experimental

Pyridinium-3-sulfonate, (1 mmol,159 mg) was dissolved in methanol (A.*R*.,99.9%) (10 ml). To the resulting clear solution was added MnCl_2_.4H_2_O (0.5 mmol, 98 mg) in methanol (10 ml). After keeping the resulting mixture in air to evaporate about half of the solvent, colourless blocks of the title compound were deposited. The crystals were isolated and washed with alcohol three times (yield 82%). Analysis found (%): C 29.38, H 2.90, N 6.89, S 15.80; C_10_H_12_MnN_2_O_8_S_2 _requires (%): C 29.47, H 2.94, N 6.87, S 15.71.

### Refinement

The H atoms of the water molecules were located in a difference map. The H atoms bonded to C atoms were placed at calculated positions and refined in a riding-model approximation [C—H = 0.93 Å and *U*_iso_(H) = 1.2*U*_eq_(C)]. The O-H distances were standardized to 0.89 Å and the H atoms of the water molecules were refined in a riding-model approximation with U_iso_(H) = 1.2*U*_eq_(O).

### Crystal data

**Table:**

[Mn(C_5_H_4_NO_3_S)_2_(H_2_O)_2_]	*F*_000_ = 414
*M**_r_* = 407.28	*D*_x_ = 1.859 Mg m^−^^3^
Monoclinic, *P*2_1_/*c*	Mo *K*α radiation λ = 0.71073 Å
Hall symbol: -P 2ybc	Cell parameters from 2334 reflections
*a* = 7.6299 (13) Å	θ = 2.7–26.4º
*b* = 13.201 (2) Å	µ = 1.24 mm^−^^1^
*c* = 7.2714 (12) Å	*T* = 294 (2) K
β = 96.516 (3)º	Block, colourless
*V* = 727.7 (2) Å^3^	0.24 × 0.22 × 0.18 mm
*Z* = 2	

### Data collection

**Table:**

Bruker SMART CCD area-detector diffractometer	1485 independent reflections
Radiation source: fine-focus sealed tube	1301 reflections with *I* > 2σ(*I*)
Monochromator: graphite	*R*_int_ = 0.022
Detector resolution: 0 pixels mm^-1^	θ_max_ = 26.4º
*T* = 298(2) K	θ_min_ = 2.7º
φ and ω scans	*h* = −8→9
Absorption correction: multi-scan(SADABS; Bruker, 1998)	*k* = −16→15
*T*_min_ = 0.755, *T*_max_ = 0.808	*l* = −9→7
4034 measured reflections	

### Refinement

**Table:**

Refinement on *F*^2^	Hydrogen site location: inferred from neighbouring sites
Least-squares matrix: full	H-atom parameters constrained
*R*[*F*^2^ > 2σ(*F*^2^)] = 0.026	*w* = 1/[σ^2^(*F*_o_^2^) + (0.0952*P*)^2^ + 1.5031*P*] where *P* = (*F*_o_^2^ + 2*F*_c_^2^)/3
*wR*(*F*^2^) = 0.069	(Δ/σ)_max_ = 0.001
*S* = 1.06	Δρ_max_ = 0.24 e Å^−^^3^
1485 reflections	Δρ_min_ = −0.37 e Å^−^^3^
106 parameters	Extinction correction: SHELXL97 (Sheldrick, 2008), Fc^*^=kFc[1+0.001xFc^2^λ^3^/sin(2θ)]^-1/4^
Primary atom site location: structure-invariant direct methods	Extinction coefficient: 0.087 (3)
Secondary atom site location: difference Fourier map	

### Special details

**Table:**

Geometry. All e.s.d.'s (except the e.s.d. in the dihedral angle between two l.s. planes) are estimated using the full covariance matrix. The cell e.s.d.'s are taken into account individually in the estimation of e.s.d.'s in distances, angles and torsion angles; correlations between e.s.d.'s in cell parameters are only used when they are defined by crystal symmetry. An approximate (isotropic) treatment of cell e.s.d.'s is used for estimating e.s.d.'s involving l.s. planes.
Refinement. Refinement of *F*^2^ against ALL reflections. The weighted *R*-factor *wR* and goodness of fit *S* are based on *F*^2^, conventional *R*-factors *R* are based on *F*, with *F* set to zero for negative *F*^2^. The threshold expression of *F*^2^ > σ(*F*^2^) is used only for calculating *R*-factors(gt) *etc*. and is not relevant to the choice of reflections for refinement. *R*-factors based on *F*^2^ are statistically about twice as large as those based on *F*, and *R*- factors based on ALL data will be even larger.

### Fractional atomic coordinates and isotropic or equivalent isotropic displacement parameters (Å^2^)

**Table:**

	*x*	*y*	*z*	*U*_iso_*/*U*_eq_	
Mn1	0.0000	1.0000	0.0000	0.01892 (13)	
S1	0.72645 (6)	0.87969 (4)	0.28519 (7)	0.01996 (14)	
O1	0.7153 (2)	0.95339 (12)	0.4310 (2)	0.0344 (4)	
O2	0.8234 (2)	0.78954 (11)	0.3466 (2)	0.0345 (4)	
O3	0.7864 (2)	0.92419 (13)	0.1200 (2)	0.0364 (4)	
O4	0.0783 (2)	1.09357 (11)	0.2404 (2)	0.0316 (4)	
H4A	0.1331	1.0735	0.3492	0.038*	
H4B	0.1133	1.1574	0.2295	0.038*	
N1	0.2084 (2)	0.88314 (12)	0.1103 (2)	0.0232 (4)	
C1	0.1706 (3)	0.78382 (15)	0.1017 (3)	0.0249 (4)	
H1	0.0547	0.7645	0.0646	0.030*	
C2	0.2946 (3)	0.70884 (16)	0.1449 (3)	0.0296 (5)	
H2	0.2627	0.6409	0.1363	0.035*	
C3	0.4668 (3)	0.73661 (15)	0.2010 (3)	0.0263 (4)	
H3	0.5534	0.6878	0.2303	0.032*	
C4	0.5079 (2)	0.83896 (15)	0.2129 (3)	0.0188 (4)	
C5	0.3761 (3)	0.90965 (14)	0.1666 (3)	0.0224 (4)	
H5	0.4047	0.9781	0.1747	0.027*	

### Atomic displacement parameters (Å^2^)

**Table:**

	*U*^11^	*U*^22^	*U*^33^	*U*^12^	*U*^13^	*U*^23^
Mn1	0.0134 (2)	0.0187 (2)	0.0237 (2)	−0.00056 (15)	−0.00157 (16)	0.00122 (16)
S1	0.0146 (2)	0.0193 (2)	0.0250 (3)	0.00040 (17)	−0.00246 (18)	0.00251 (18)
O1	0.0305 (8)	0.0318 (9)	0.0386 (9)	0.0022 (7)	−0.0057 (7)	−0.0107 (7)
O2	0.0252 (8)	0.0247 (8)	0.0499 (10)	0.0067 (6)	−0.0114 (7)	0.0029 (7)
O3	0.0228 (8)	0.0506 (10)	0.0356 (9)	−0.0106 (7)	0.0022 (6)	0.0125 (8)
O4	0.0398 (9)	0.0249 (8)	0.0281 (8)	−0.0042 (7)	−0.0054 (7)	−0.0024 (6)
N1	0.0170 (8)	0.0215 (8)	0.0302 (9)	−0.0009 (7)	−0.0012 (7)	0.0025 (7)
C1	0.0186 (10)	0.0253 (10)	0.0298 (11)	−0.0038 (8)	−0.0014 (8)	0.0006 (9)
C2	0.0281 (12)	0.0184 (10)	0.0416 (13)	−0.0043 (8)	0.0011 (10)	−0.0005 (9)
C3	0.0213 (10)	0.0182 (10)	0.0393 (12)	0.0042 (8)	0.0025 (9)	0.0039 (8)
C4	0.0152 (9)	0.0206 (10)	0.0205 (9)	−0.0006 (7)	0.0009 (7)	0.0013 (7)
C5	0.0186 (10)	0.0156 (9)	0.0322 (11)	−0.0011 (7)	−0.0009 (8)	0.0012 (8)

### Geometric parameters (Å, °)

**Table:**

Mn1—O4	2.1681 (15)	N1—C5	1.345 (3)
Mn1—O3^i^	2.1773 (15)	C1—C2	1.381 (3)
Mn1—N1	2.2937 (16)	C1—H1	0.9300
S1—O2	1.4449 (15)	C2—C3	1.380 (3)
S1—O1	1.4489 (16)	C2—H2	0.9300
S1—O3	1.4570 (16)	C3—C4	1.388 (3)
S1—C4	1.7737 (19)	C3—H3	0.9300
O4—H4A	0.8922	C4—C5	1.385 (3)
O4—H4B	0.8897	C5—H5	0.9300
N1—C1	1.342 (3)		
			
O4—Mn1—O4^ii^	180.0	C1—N1—C5	117.40 (17)
O4—Mn1—O3^i^	95.12 (6)	C1—N1—Mn1	120.26 (13)
O4^ii^—Mn1—O3^i^	84.88 (6)	C5—N1—Mn1	121.96 (13)
O3^iii^—Mn1—O3^i^	180.0	N1—C1—C2	123.47 (18)
O4—Mn1—N1	89.13 (6)	N1—C1—H1	118.3
O4^ii^—Mn1—N1	90.87 (6)	C2—C1—H1	118.3
O3^iii^—Mn1—N1	85.90 (6)	C1—C2—C3	118.80 (19)
O3^i^—Mn1—N1	94.09 (6)	C1—C2—H2	120.6
N1^ii^—Mn1—N1	180.00 (6)	C3—C2—H2	120.6
O2—S1—O1	113.41 (10)	C2—C3—C4	118.54 (18)
O2—S1—O3	112.91 (10)	C2—C3—H3	120.7
O1—S1—O3	112.51 (10)	C4—C3—H3	120.7
O2—S1—C4	105.84 (9)	C5—C4—C3	119.22 (18)
O1—S1—C4	106.81 (9)	C5—C4—S1	119.99 (15)
O3—S1—C4	104.50 (9)	C3—C4—S1	120.79 (15)
S1—O3—Mn1^iv^	146.62 (10)	N1—C5—C4	122.57 (18)
Mn1—O4—H4A	127.1	N1—C5—H5	118.7
Mn1—O4—H4B	121.7	C4—C5—H5	118.7
H4A—O4—H4B	104.2		
			
O2—S1—O3—Mn1^iv^	−66.4 (2)	C1—C2—C3—C4	0.4 (3)
O1—S1—O3—Mn1^iv^	63.6 (2)	C2—C3—C4—C5	−0.6 (3)
C4—S1—O3—Mn1^iv^	179.06 (19)	C2—C3—C4—S1	179.69 (16)
O4—Mn1—N1—C1	137.80 (16)	O2—S1—C4—C5	172.26 (16)
O4^ii^—Mn1—N1—C1	−42.20 (16)	O1—S1—C4—C5	51.12 (18)
O3^iii^—Mn1—N1—C1	−137.26 (16)	O3—S1—C4—C5	−68.31 (18)
O3^i^—Mn1—N1—C1	42.73 (16)	O2—S1—C4—C3	−8.1 (2)
O4—Mn1—N1—C5	−49.49 (16)	O1—S1—C4—C3	−129.20 (18)
O4^ii^—Mn1—N1—C5	130.51 (16)	O3—S1—C4—C3	111.38 (18)
O3^iii^—Mn1—N1—C5	35.44 (16)	C1—N1—C5—C4	0.7 (3)
O3^i^—Mn1—N1—C5	−144.56 (16)	Mn1—N1—C5—C4	−172.23 (14)
C5—N1—C1—C2	−0.9 (3)	C3—C4—C5—N1	0.1 (3)
Mn1—N1—C1—C2	172.13 (16)	S1—C4—C5—N1	179.77 (16)
N1—C1—C2—C3	0.4 (3)		

Symmetry codes: (i) *x*−1, *y*, *z*; (ii) −*x*, −*y*+2, −*z*; (iii) −*x*+1, −*y*+2, −*z*; (iv) *x*+1, *y*, *z*.

### Hydrogen-bond geometry (Å, °)

**Table:**

*D*—H···*A*	*D*—H	H···*A*	*D*···*A*	*D*—H···*A*
O4—H4B···O2^v^	0.89	1.91	2.786 (2)	168
O4—H4A···O1^vi^	0.89	1.90	2.778 (2)	169

Symmetry codes: (v) −*x*+1, *y*+1/2, −*z*+1/2; (vi) −*x*+1, −*y*+2, −*z*+1.
